# The novel compound Sul-121 inhibits airway inflammation and hyperresponsiveness in experimental models of chronic obstructive pulmonary disease

**DOI:** 10.1038/srep26928

**Published:** 2016-05-27

**Authors:** Bing Han, Wilfred J. Poppinga, Haoxiao Zuo, Annet B. Zuidhof, I. Sophie T. Bos, Marieke Smit, Pieter Vogelaar, Guido Krenning, Robert H. Henning, Harm Maarsingh, Andrew J. Halayko, Bernard van Vliet, Stef Stienstra, Adrianus Cornelis van der Graaf, Herman Meurs, Martina Schmidt

**Affiliations:** 1 University of Groningen, Department of Molecular Pharmacology, Groningen, the Netherlands; 2 GRIAC research institute, University of Groningen, University Medical Center Groningen, the Netherlands; 3 Sulfateq B.V. Groningen, the Netherlands; 4 University of Groningen, University Medical Center Groningen, Dept. Pathology and Medical Biology, Laboratory for Cardiovascular Regenerative Medicine, Groningen, the Netherlands; 5 University of Groningen, Department of Clinical Pharmacy and Pharmacology, Groningen, the Netherlands; 6 Palm Beach Atlantic University, Lloyd L. Gregory School of Pharmacy, Department of Pharmaceutical Sciences, West Palm Beach, FL, USA; 7 Department of Physiology and Pathophysiology, University of Manitoba, Winnipeg, Manitoba, Canada

## Abstract

COPD is characterized by persistent airflow limitation, neutrophilia and oxidative stress from endogenous and exogenous insults. Current COPD therapy involving anticholinergics, β_2_-adrenoceptor agonists and/or corticosteroids, do not specifically target oxidative stress, nor do they reduce chronic pulmonary inflammation and disease progression in all patients. Here, we explore the effects of Sul-121, a novel compound with anti-oxidative capacity, on hyperresponsiveness (AHR) and inflammation in experimental models of COPD. Using a guinea pig model of lipopolysaccharide (LPS)-induced neutrophilia, we demonstrated that Sul-121 inhalation dose-dependently prevented LPS-induced airway neutrophilia (up to ~60%) and AHR (up to ~90%). Non-cartilaginous airways neutrophilia was inversely correlated with blood H_2_S, and LPS-induced attenuation of blood H_2_S (~60%) was prevented by Sul-121. Concomitantly, Sul-121 prevented LPS-induced production of the oxidative stress marker, malondialdehyde by ~80%. In immortalized human airway smooth muscle (ASM) cells, Sul-121 dose-dependently prevented cigarette smoke extract-induced IL-8 release parallel with inhibition of nuclear translocation of the NF-κB subunit, p65 (each ~90%). Sul-121 also diminished cellular reactive oxygen species production in ASM cells, and inhibited nuclear translocation of the anti-oxidative response regulator, Nrf2. Our data show that Sul-121 effectively inhibits airway inflammation and AHR in experimental COPD models, prospectively through inhibition of oxidative stress.

Chronic obstructive pulmonary disease (COPD) is one of the leading causes of death worldwide, which results a significant economic and social burden^1^. It is characterized by persistent and progressive airflow limitation and prominent neutrophilic airway inflammation^1^,^2^. Currently, reduction of COPD symptoms is achieved mainly with (a combination of) anticholinergics, β_2_-adrenoceptor agonists and glucocorticosteroids^1^. However, the current medications fail to reduce the progression of COPD and have even been associated with fatal side effects^3^,^4^,^5^,^6^.

Oxidative stress, caused by an anti-oxidant/oxidant imbalance that leads to increased generation of reactive oxygen species (ROS), is believed to play an important role in the pathogenesis of COPD^7^. ROS derived from exogenous (environmental pollution, cigarette smoke) or endogenous (inflammatory cells, such as neutrophils) insult, promote the activation of the pro-inflammatory transcription factor nuclear factor (NF)-κB in structural lung cells including airway smooth muscle cells^8^. Activation of NF-κB results in increased secretion of inflammatory cytokines such as interleukin (IL)-8^9^,^10^,^11^, which recruit inflammatory cells, including neutrophils^5^. Moreover, ROS trigger the peroxidative breakdown of lipids, a process implicated in lung injuries due to increased airway epithelial permeability^12^. ROS have also been implicated as an important cause of steroid resistance in COPD^13^. Targeting oxidative stress might be a beneficial approach for the management of COPD. Although evidence for clear clinical benefit is currently lacking, many anti-oxidative strategies using anti-oxidants or pharmacological agents have shown promising effects in COPD^7^,^14^.

Due to its anti-oxidative capacities, the gasotransmitter hydrogen sulfate (H_2_S), synthesized by enzymes such as cystathionine β synthetase (CBS)^15^, has been proposed as a potential COPD treatment^16^. By contrast, some studies indicated that its redox potential may provoke pro-inflammatory responses, for instance, oxidative stress originated from activated neutrophils can convert H_2_S to sulfite^17^, which is considered an inflammatory mediator in airway diseases^18^. Therefore, the usage of H_2_S or its donors to treat COPD is still under debate.

One mechanism that could underpin an anti-oxidant response to H_2_S is the activation of nuclear factor erythroid 2-related factor 2 (Nrf2), a transcription factor that increases the expression of anti-oxidant proteins in structural airway cells, including smooth muscle cells^12^,^19^. Indeed, H_2_S is able to activate Nrf2 both *in vivo* in mouse models of lung injury and *in vitro* in embryonic fibroblasts^20^,^21^,^22^.

Recently, we developed a novel class of pharmacological compounds of which Sul-121 (6-hydroxy-2,5,7,8-tetramethylchroman-2-yl (piperazin-1-yl) methanone, Fig. 1A) is one of its leads with promising cell protective effects due to anti-oxidant capacities^23^. In the present study, we explored the pharmacological potential of Sul-121 in *in vitro* and *in vivo* experimental models of COPD. We report that Sul-121 prevents lipopolysaccharide (LPS)-induced neutrophilia, hyperresponsiveness (AHR) and oxidative stress in guinea pigs. In addition, Sul-121 reduces the cigarette smoke-induced release of IL-8 in cultured human airway smooth muscle (ASM) cells, which is accompanied by a reduction in cellular ROS production and nuclear translocation of Nrf2.

## Results

### Effects of Sul-121 on LPS-induced AHR

**Figure 1:**
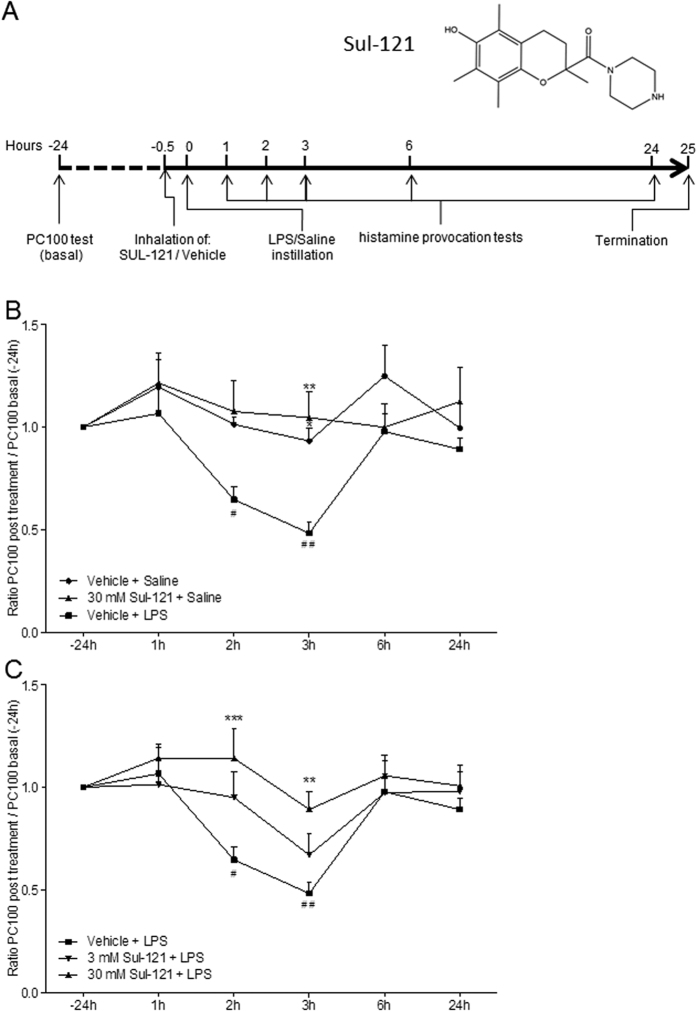
Effects of Sul-121 on LPS-induced airway responsiveness in guinea pigs. Guinea pigs were intranasally instilled with LPS (5 mg/ml in saline, 300 μl; t = 0 h) to induce AHR, or with saline (control) as outlined in the Material and Methods. At 30 min before LPS or saline instillation, animals were treated by inhalation of aerosolized vehicle or Sul-121 (3 or 30 mM nebulizer concentration). Inset, structure of Sul-121 (6-hydroxy-2,5,7,8-tetramethylchroman-2-yl (piperazin-1-yl) methanone. At 25 h after LPS challenge, animals were terminated (**A**). Airway responsiveness to histamine in the different treatment groups was assessed by determining the provocation concentration of histamine causing a 100% increase in P_pl_ (PC100). Data are expressed as ratio between the PC100-value at the different time points over the PC100-value at baseline (t = −24 h), with a value of 1 representing normoresponsiveness (**B,C**). N = 4–6 animals per group. (**C**) ^#^p < 0.05, ^##^p < 0.01, compared with baseline (t = −24 h); one way ANOVA repeated measurement with bonferroni’s multiple comparison tests. (**B,C**) *p < 0.05, **p < 0.01, ***p < 0.001 compared with the LPS control group at the same time point; two way ANOVA with bonferroni post-tests.

In line with earlier reports^24^,^25^, we observed increased airway responsiveness to histamine 2 and 3 hours after LPS instillation, with PC100 values (the provocation concentration of histamine causing a 100% increase in pleural pressure (P_pl_)) at these time points significantly lower than that for LPS-naive animals that received only saline instillation (Fig. 1B). Although inhaled Sul-121 did not affect airway responsiveness in LPS-naive control animals (Fig. 1B), it did prevent LPS-induced AHR in a dose-dependent manner up to 90% (Fig. 1C). At 30 mM (nebulizer concentration), Sul-121 almost fully prevented LPS-induced AHR (0.89 ± 0.09, p < 0.01; Fig. 1C). In line with our observation that Sul-121 did not affect airway responsiveness in LPS-naive, saline-challenged animals, it also did not affect methacholine-induced contraction of bovine tracheal smooth muscle strips (Supplementary Fig. 1A). This finding strongly suggests that Sul-121 does not have a direct effect on airway smooth muscle tone.

### Effects of Sul-121 on LPS-induced Neutrophilia in BALF and Airway Tissue

**Figure 2:**
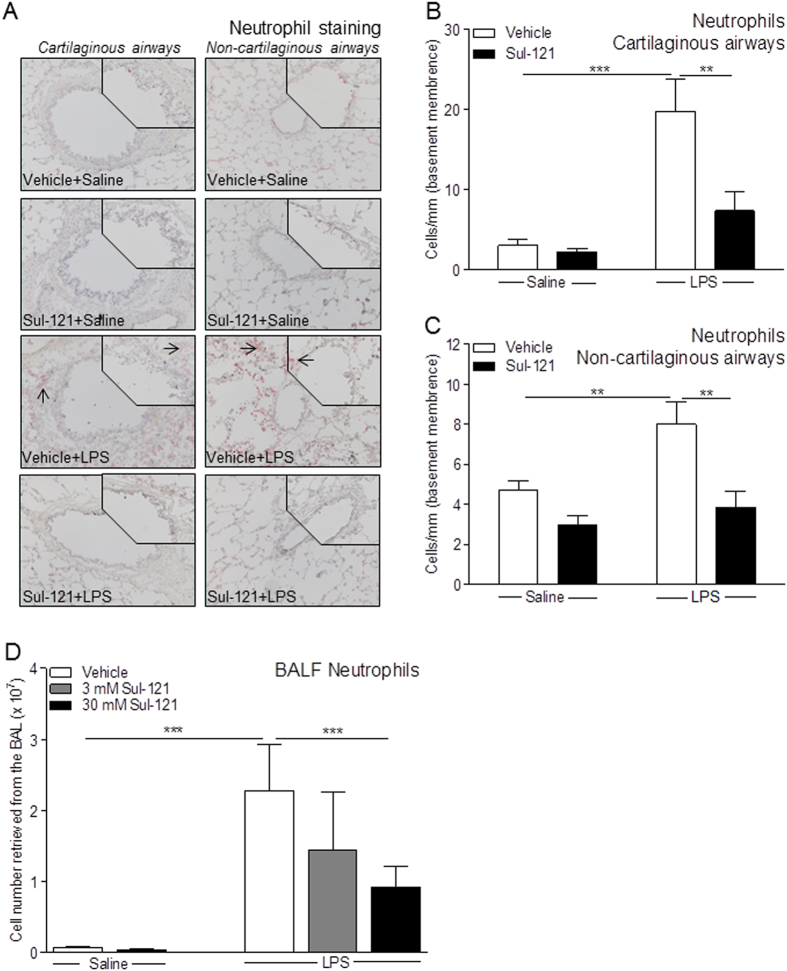
Effects of Sul-121 on LPS-induced neutrophilia in BALF, blood and airway tissue. Guinea pigs were treated for 3 min by inhalation of vehicle or Sul-121 (3 or 30 mM nebulizer concentration) 30 min before intranasal instillation of 300 μl saline or LPS (5 mg/ml) at 0 h. After animal termination at 25 h, BAL were performed, and blood and lungs were collected for further analysis. Neutrophils were stained with TNAP (red staining, arrows) in transverse frozen cross-sections of the lung. Representative images are shown (**A**), magnification: 10X, inset, 20X). Neutrophils in cartilaginous (**B**) and non-cartilaginous airways (**C**) on sections were quantified in the adventitia and sub-mucosa, and expressed as the number of positively stained cells per mm basement membrane length. Neutrophil numbers were determined in BALF (**D**) and blood (**E**). N = 4–6. (**B,C,D**) **p < 0.01, ***p < 0.001; two way ANOVA with bonferroni post-tests.

As expected^24^,^25^,^26^, LPS challenge significantly increased the number of lung neutrophils in both cartilaginous (from 3.0 ± 0.8 to 19.8 ± 4.2 cells/mm basement membrane) and non-cartilaginous airways (from 4.7 ± 0.5 to 8.008 ± 1.099 cells/mm basement membrane), which was largely prevented by 30 mM Sul-121 in both airway categories (cartilaginous airways: 7.340 ± 2.402 cells/mm basement membrane; non-cartilaginous airways: 3.834 ± 0.824 cells/mm basement membrane; p < 0.01 both; Fig. 2A–C). Similar to the neutrophil changes in the airways, LPS induced a significant increase of neutrophils in bronchoalveolar lavage (BAL) fluids (BALFs) (from 0.07 ± 0.02 to 2.28 ± 0.66 × 10^7^ cell number retrieved from the BAL, Fig. 2D). Inhalation of Sul-121 dose-dependently prevented LPS-induced neutrophilia by up to 60% using 30 mM Sul-121 (to 0.91 ± 0.30 × 10^7^ cell number retrieved from the BAL; p < 0.001; Fig. 2D). Sul-121 was without effect on basal neutrophil numbers in the lungs of saline challenged, LPS-naive animals (Fig. 2D).

### Blood H_2_S level and Lung CBS expression

**Figure 3:**
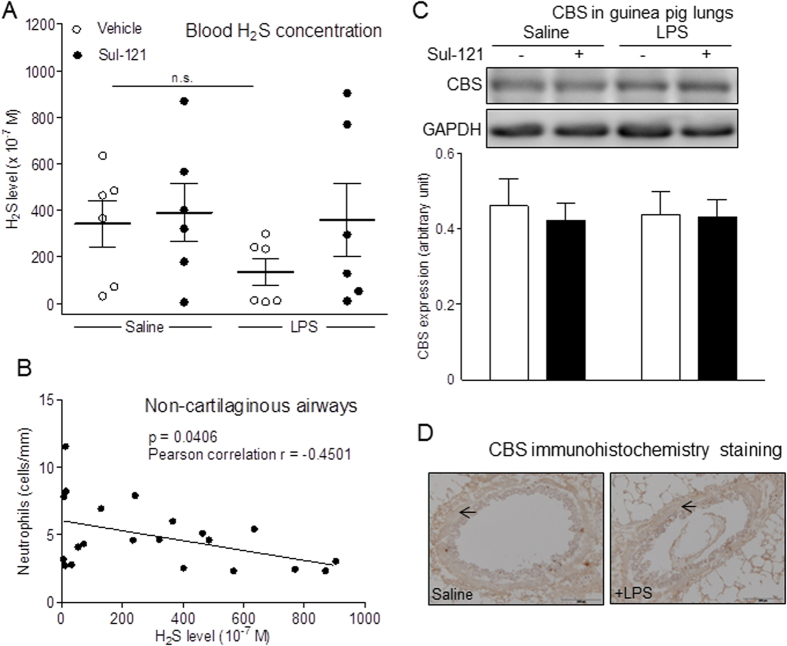
Effects of Sul-121 on blood H_2_S level and lung CBS expression. Guinea pigs were treated for 3 min by inhalation of vehicle or 30 mM Sul-121 at 30 min before intranasal instillation of 300 μl saline or LPS (5 mg/ml) at 0 h. After animal termination at 25 h, blood/serum was collected and H_2_S levels were measured (**A**). Correlations between serum H_2_S levels and neutrophil numbers in non-cartilaginous (**B**) were determined. Western analysis of CBS protein expression was performed in lung homogenates. Cropped images are shown. Full-length blots are presented in Supplementary Fig. 2. Gels have been run under the same experimental condition (**C**). CBS expression (yellow brown staining, arrows) was determined using immunohistochemistry in transverse frozen cross-sections of the lung. Representative images are shown (**D**). N = 4–6. A: n.s: not significant versus saline vehicle.

H_2_S may have a protective role in inflammatory airway diseases^16^. LPS challenge tended to decrease levels of serum H_2_S (from 342 ± 99 to 134 ± 56 × 10^−7^ M), an effect prevented in guinea pigs pretreated with 30 mM Sul-121 (360 ± 157 × 10^−7^ M; Fig. 3A). As shown in Fig. 3B, in both LPS-naive, saline challenged and LPS challenged animals we found an inverse correlation between serum H_2_S levels and the number of neutrophils in non-cartilaginous airways (p = 0.041, r = 0.45). Neither immunohistochemistry nor western blotting revealed any difference in the abundance of the H_2_S producing enzyme, CBS^15^, in the lungs of animals in any of our study groups (Fig. 3C,D; Supplementary Fig. 2). Overall, our data suggest that Sul-121 protects concomitant loss of serum H_2_S and against the accumulation of lung neutrophils that are otherwise induced by LPS challenge.

### Lung Nrf2 expression and MDA Levels

**Figure 4:**
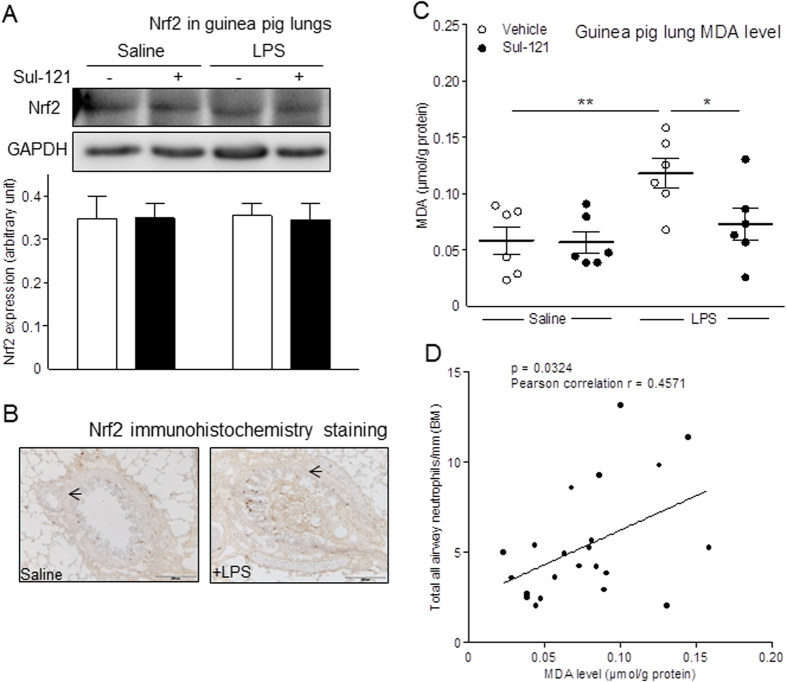
Effects of Sul-121 on lung Nrf2 expression and MDA level in lung homogenates. Guinea pigs were treated for 3 min by inhalation of vehicle or 30 mM Sul-121 at 30 min before intranasal instillation of 300 μl saline or LPS (5 mg/ml) at 0 h followed by animal termination on 25 hours. Western analysis of Nrf2 protein expression was performed in lung homogenates. Cropped images are shown. Full-length blots are presented in Supplementary Fig. 3. Gels have been run under the same experimental condition (**A**). Nrf2 expression (yellow brown staining, arrows) was determined using immunohistochemistry in transverse frozen cross-sections of the lung. Representative images are shown (**B**). MDA levels were measured in lung homogenates (**C**). Correlation between lung MDA concentration and total airway neutrophil numbers was performed (D). N = 4–6. (**C**) *p < 0.05, **p < 0.01; two way ANOVA with bonferroni post-tests.

Neither LPS nor Sul-121, alone or in combination altered the expression of the anti-oxidant transcription factor Nrf2 expression in lung homogenates (Fig. 4A; Supplementary Fig. 3) or lung sections (Fig. 4B). Polyunsaturated lipids can be degraded under oxidative stress leading to the formation of MDA^27^. We therefore analyzed oxidative stress in lung tissue by measuring total MDA levels. LPS induced a 2-fold increase in MDA abundance (from 0.06 ± 0.01 to 0.12 ± 0.01 μmol/g protein; p < 0.01), confirming that oxidative stress had developed, and 30 mM Sul-121 fully prevented the LPS-induced MDA in lungs (0.07 ± 0.01 μmol/g protein; p < 0.05; Fig. 4C). Moreover, we found a positive correlation between lung MDA level and airway neutrophil number (p = 0.0324, r = 0.457; Fig. 4D). Taken together, these findings indicate that Sul-121 may - at least partially, exert its protective effects in the lung by normalization of LPS-induced oxidative stress.

### IL-8 Release and p65 Nuclear Translocation in hTERT Cells

**Figure 5:**
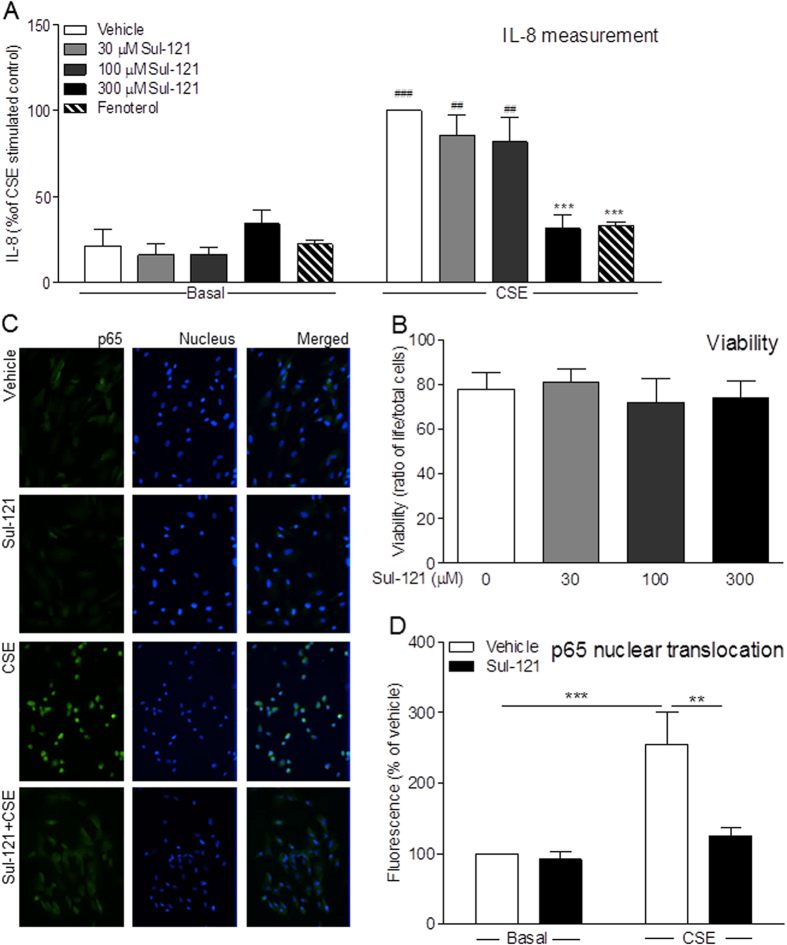
Effects of Sul-121 on IL-8 release and p65 nuclear translocation in ASM cells. Immortalized human ASM cells were incubated with Sul-121 (10–300 μM) in the absence of presence of 15% CSE for 24 h. Fenoterol (1 μM) served as positive control. IL-8 concentrations in supernatants were measured by ELISA (**A**). Cells were trypsinized for trypan blue cell counting to determine cell viability (**B**). For immunofluorescence of p65 (**C**), hTERT cells were incubated with 300 μM Sul-121 in the absence and or presence of 15% CSE for 2 hours. Representative images are shown. Images were quantified by Image J 1.48v (**D**). (**A**,**B**) N = 7–24; ***p < 0.001 compared to CSE vehicle; two way ANOVA with Bonferroni post-tests; ^##^p < 0.01, ^###^p < 0.00, compared to corresponding basal IL-8 release, two way ANOVA with Bonferroni post-tests. D: N = 5; **p < 0.01, **p < 0.001; two way ANOVA with Bonferroni post-tests.

The mechanisms by which Sul-121 may decrease airway neutrophilia were further studied *in vitro* using human ASM cells, which we have previously shown, and confirm here, to be capable of expressing and releasing abundant IL-8 in response to challenge with CSE by about 5-fold above basal (34 ± 13 pg/ml)^9^,^10^ (Fig. 5A). Treatment of cells with Sul-121 dose-dependently reduced 15% CSE-induced IL-8 release up to 90%, with 300 μM Sul-121 almost fully abrogating IL-8 release (p < 0.001); an effect on par with that elicited by 1 μM fenoterol, a first line COPD therapeutic^28^ (Fig. 5A). Cell viability was not compromised by any concentrations of Sul-121 (Fig. 5B). CSE exposure significantly increased nuclear translocation of the NF-κB subunit, p65, an effect that was fully prevented by Sul-121 (from 255 ± 46 to 126 ± 12 fluorescence % of vehicle; p < 0.01; Fig. 5C,D).

### ROS production and Nrf2 nuclear translocation in ASM cells

**Figure 6:**
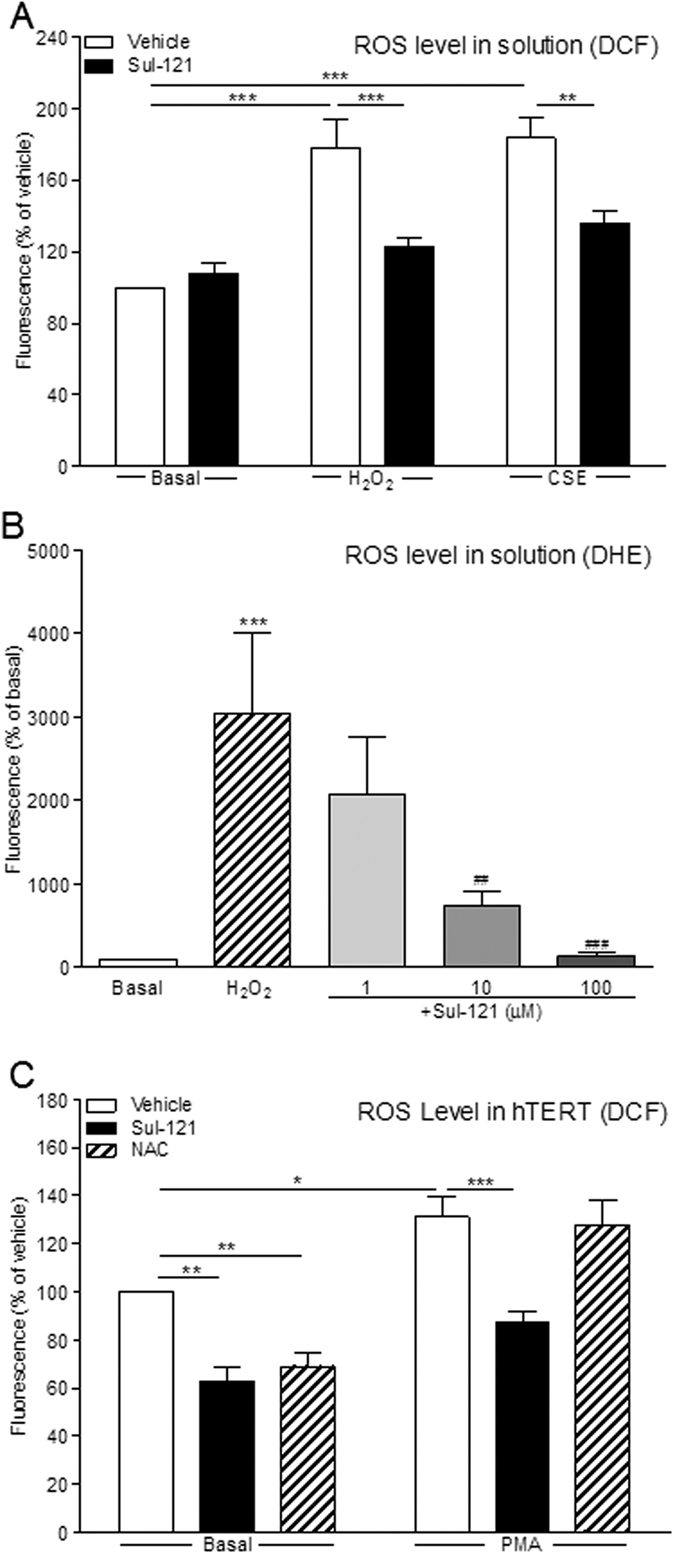
Effects of Sul-121 on ROS production. Under cell-free condition, carboxy-H2DCF-DA (0.1 μM) was incubated for 40 min with 300 μM Sul-121 in the absence and presence of H_2_O_2_ (2 mM) or CSE (15%). ROS was measured by the intensity of DCF emission (**A**). Alternatively, DHE (5 μM) was incubated for 1 hour with Sul-121 (1–100 μM) in the absence and presence of H_2_O_2_ (5 μM). ROS was measured by the intensity of DHE emission (**B**). Immortalized human ASM cells were incubated with 300 μM Sul-121 in the absence and presence of PMA (0.1 μM) for 2 hours. 10 mM NAC served as positive control. After removal of the supernatant, cells were incubated with carboxy-H2DCFDA (0.1 μM) for 1 h. ROS was measured by the intensity of DCF emission (**C**). (**A**) N = 12; **p < 0.01, ***p < 0.001; two way ANOVA with Bonferroni post-tests. (**B**) N = 3, ***p < 0.001, compared to Basal; one way ANOVA with Bonferroni post-tests. ^##^p < 0.01, ^###^p < 0.001, compared to H_2_O_2_ treatment; one way ANOVA with Bonferroni post-tests. (**C**) N = 4; *p < 0.05, **p < 0.01, ***p < 0.001; two way ANOVA with Bonferroni post-tests.

Sul-121 (300 μM) reduced ROS levels in 2 mM H_2_O_2_ (from 177 ± 17 to 123 ± 4.9 fluorescence % of vehicle; p < 0.001) or 15% CSE (from 183 ± 12 to 136 ± 6.9 fluorescence % of vehicle; p < 0.001) solutions under cell-free condition (Fig. 6A). In addition, Sul-121 completely abolished in a dose-dependent manner ROS levels in 5 μM H_2_O_2_ (Fig. 6B), indicating that Sul-121 normalizes exogenous ROS in all experimental conditions. Thus the effects of Sul-121 on cell responses are likely due to direct effects on cellular oxidative stress and associated cell responses. Indeed, Sul-121 reduced endogenous ROS production by human ASM cells to the same degree as the broadly effective ROS inhibitor, 10 mM N-acetyl-L-cysteine (NAC; to 62 ± 6.4 and 69 ± 5.7 fluorescence % of vehicle, respectively; Fig. 6C). However, Sul-121, but not NAC, significantly reduced 0.1 μM phorbol 12-myristate 13-acetate (PMA)-induced ROS production by ASM cells (from 132 ± 8.3 to 88 ± 4.4 fluorescence % of vehicle; p < 0.001; Fig. 6C).

**Figure 7:**
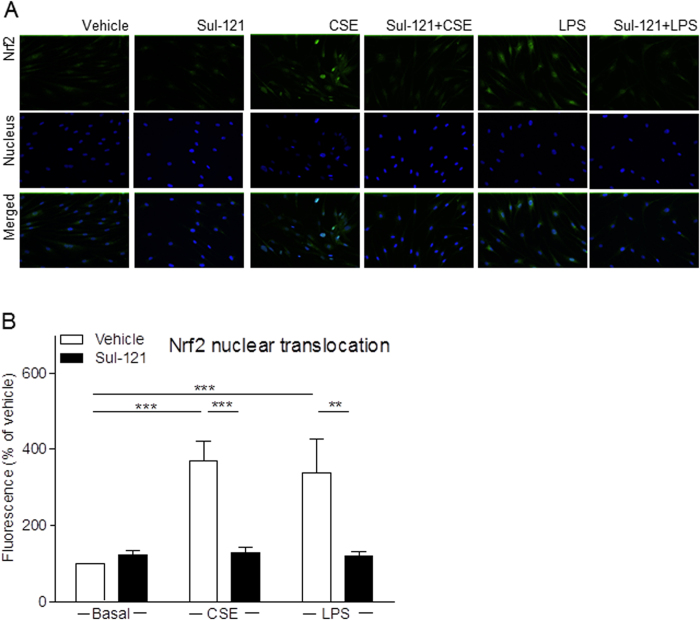
Effects of Sul-121 on Nrf2 nuclear translocation in ASM cells. For immunofluorescence of Nrf2, immortalized human ASM cells were incubated with 300 μM Sul-121 in the absence and or presence of 15% CSE or 10 μg/ml LPS for 2 h. Representative images are shown (**A**). Images were quantified by Image J 1.48v (**B**). N = 4–9. (**B**) **p < 0.01, ***p < 0.001; two way ANOVA with Bonferroni post-tests.

We found that 15% CSE and 10 μg/ml LPS exposure significantly increased the nuclear translocation of the anti-oxidant transcription factor Nrf2 (CSE: 368 ± 52 fluorescence % of vehicle, p < 0.001; LPS: 338 ± 88 fluorescence % of vehicle, p < 0.001; Fig. 7A,B) as measured by immunofluorescence. Notably, 300 μM Sul-121 completely prevented Nrf2 translocation to the nucleus induced by both CSE (127 ± 16 fluorescence % of vehicle; p < 0.001; Fig. 7A,B) and LPS (118 ± 13 fluorescence % of vehicle; p < 0.01; Fig. 7A,B).

## Discussion

COPD is characterized by persistent and progressive airflow limitation associated with chronic pulmonary inflammation^1^. Although often underestimated in clinical assessment, AHR is an important characteristic of COPD^29^ that associates with accelerated lung function decline^30^,^31^. There is a strong association between AHR and accumulation of lung neutrophils^29^, which are associated with production of ROS and a number of potent pro-inflammatory cytokines^3^,^32^. In our present study, using a guinea pig model of LPS-induced neutrophilia that mimics that in COPD and promotes the development of AHR, we show that Sul-121 dose-dependently prevents AHR induced by intranasal instillation of LPS *in vivo*. The acute LPS-challenge model we employ induces marked accumulation of lung neutrophils, a feature that correlates strongly with clinical outcomes^2^,^33^,^34^. Here we report from *in vitro* and *in vivo* studies that though Sul-121 has no direct bronchodilatory effects, it does strongly inhibit the development of AHR, primarily by inhibiting LPS-induced lung inflammation. Indeed, inhaled Sul-121 prevents LPS-induced lung neutrophilia, confirmed in BALF and in both cartilaginous and non-cartilaginous airways, as well as the induction of markers of oxidative stress in the lungs, demonstrating that Sul-121 possesses significant anti-oxidant and anti-inflammatory properties.

As a potent neutrophil chemoattractant and activator^2^, levels of IL-8 in the lungs are strongly correlated with neutrophil number in COPD patients^35^,^36^. We report that Sul-121 dose-dependently reduces CSE-induced IL-8 release from human ASM cells *in vitro*, potentially explaining the capacity for inhaled Sul-121 to block LPS-induced lung neutrophilia *in vivo*. NF-κB is involved in the transcription of a variety of pro-inflammatory genes, including IL-8, and is activated by CSE^9^,^10^. The activation of NF-κB is associated with the translocation of its p65 subunit to the nucleus, subsequently triggering transcription of inflammatory cytokines and chemokines^37^. We show that Sul-121 pre-treatment effectively prevents CSE-induced p65 nuclear translocation in human ASM cells, an effect that parallels suppression of CSE-induced IL-8 release. Taken together, our current findings indicate that Sul-121 may prevent airway neutrophilic inflammation by decreasing IL-8 release upon inhibition of NF-κB activation and subsequent nuclear translocation.

Oxidative stress plays a central role in inflammatory responses in COPD^7^. NF-κB can be activated by oxidative stress, leading to downstream inflammatory responses^38^. Therefore, the anti-inflammatory effect of Sul-121 could be explained by an anti-oxidant effect. Nrf2 is a nuclear factor that controls cellular anti-oxidative responses^39^. Under favorable physiological conditions, the Nrf2 activity is suppressed by Keap1, a cytosolic protein binding partner that prevents Nrf2 nuclear translocation. Under conditions of oxidative stress, Keap1 dissociates from, and permits nuclear translocation of Nrf2^40^. Subsequently, Nrf2-induced transcription of anti-oxidant genes initiates adaptive responses that can counteract oxidative stress^39^. We now report that CSE, as well as LPS, exposure of human ASM cells induces Nrf2 nucleus translocation, and that Sul-121 significantly decreases Nrf2 nucleus translocation induced by both CSE and LPS. Although these findings at first seem contradictory, they actually do support an anti-oxidant role for Sul-121 as they diminish the requirement for endogenous pathways to increase the transcription of anti-oxidative stress genes. In support of an anti-oxidant property for Sul-121, we report that it significantly reduces both exogenous ROS levels in pro-oxidant treatments (e.g. CSE and H_2_O_2_), as well as endogenous ROS produced by ASM cells in response to PMA. Taken together, our findings support a hypothesis that Sul-121 exerts anti-inflammatory effects through normalization of oxidative stress.

Accordingly, LPS-induced elevation of MDA, a product of peroxidative breakdown of polyunsaturated fatty acids^27^, is effectively reduced in guinea pigs pretreated with Sul-121. Importantly, lung MDA levels correlate with lung neutrophil infiltration induced by LPS *in vivo*. The anti-oxidant properties of Sul-121 may explain, at least in part, its capacity in the present study to suppress LPS-induced AHR, as oxidative stress has been directly implicated to underpin pathogenesis leading to decreased lung function^41^,^42^. In addition, peroxidative breakdown of polyunsaturated fatty acids contributes to impairment of the epithelial integrity^43^,^44^, which can contribute to increased transmigration of neutrophils to the airway lumen^45^. Since CSE has been shown to impair airway epithelial integrity *in vitro*^46^,^47^, it is tempting to speculate that the prevention of LPS-induced airway neutrophilia by Sul-121 may be associated with maintenance of epithelial integrity due to its anti-oxidative properties. Our work supports future studies to fully investigate this possibility.

Notably, H_2_S reportedly protects against LPS-induced lung injury^16^. Several studies have focused on the effects of exogenous H_2_S donors on inflammation^20^,^48^,^49^. Nonetheless, little is known about the relationship between airway inflammation and endogenous H_2_S production. Serum H_2_S levels may be decreased in COPD patients during acute exacerbation^50^, and patients requiring antibiotics due to lower respiratory tract infections exhibit significantly reduced serum H_2_S levels compared subjects not requiring antibiotics^51^. In line with these findings, we report that LPS challenge induces a trend towards reduced serum H_2_S, and serum H_2_S levels negatively correlate with neutrophil number in non-cartilaginous (p = 0.0406). The oxidation of blood H_2_S to its pro-inflammatory sulfite form occurs during oxidative stress generated upon exposure of neutrophils to LPS^17^. Therefore, we speculate that LPS-induced oxidative stress leads to loss of serum H_2_S in the guinea pig model we have employed. Interestingly, resistance to corticosteroid therapies in COPD patients has been attributed to the imbalance of acetylation-deacetylation states of histones due to the impact of oxidative stress on histone deacetylase^13^,^52^. Thus, it would be interesting to study whether Sul-121 prevents or reverses corticosteroid resistance in experimental models of COPD.

In conclusion, we show that Sul-121 reduces neutrophilic inflammation and AHR in an LPS-induced experimental model of COPD *in vivo*, probably due to the reduction of oxidative stress as well as inhibition of NF-κB and Nrf2 activation. These findings support future work to determine the potential for Sul-121 as a candidate for treatment of COPD.

## Methods

### Animals

Outbred male, specified pathogen-free Dunkin Hartley guinea pigs (Harlan, Heathfield, UK) weighing 350–450 g were used. Guinea pigs were randomly divided into indicated experimental groups with 4-6 guinea pigs per group. All *in vivo* protocols described in this study were approved by the University of Groningen (Groningen, The Netherlands) Committee for Animal Experimentation. All the methods were carried out in accordance with the approved guidelines.

### Animal Model of COPD

Guinea pigs were intranasally instilled with LPS (Sigma, L-2880) to induce neutrophilic airway inflammation and AHR^24^,^25^,^26^. Guinea pigs were held in upright position while 300 μl LPS (5 mg/ml in sterile saline) was slowly instilled intranasally and kept in the upright position for an additional 2 min to allow sufficient spreading of the fluid throughout the airways. Control animals were instilled with 300 μl sterile saline.

### Experimental *In Vivo* Protocols

As shown in Fig. 1A, 30 min before LPS or saline instillation animals were treated by inhalation of aerosolized vehicle (2% dimethyl sulfoxide and 0.2% Tween80 in saline) or Sul-121 solutions (3 or 30 mM, nebulizer concentrations) for 3 min in a 9-liter Perspex cage^53^. A DeVilbiss nebulizer (type 646) driven by an airflow of 8 l/min provided the aerosol with an output of 0.33 ml/min. Airway responsiveness to histamine was measured 24 hours before (basal) and 1, 2, 3, 6 and 24 hours after LPS/saline instillation by lung function measurements as described below. At 25 hours after LPS challenge, BAL was performed to assess inflammatory cell infiltration in the airways.

In a separate protocol, guinea pigs inhaled Sul-121 (30 mM, 3 min) or vehicle 30 min before LPS or saline challenge, followed by blood and lung tissue collection at 25 hours later. Blood was collected by heart puncture and stored in EDTA coated tubes. Neutrophil numbers were determined in blood. Serum was obtained by centrifuging (2000 rcf, 10 min) to quantify levels of Sul-121 and H_2_S. Lung tissue was snap frozen and used for histology (neutrophils) and immunohistochemistry (CBS, Nrf2) using transverse frozen cross-sections (5 μm) of the upper right lung lobe, and for Western blotting (CBS, Nrf2) and malondialdehyde (MDA) measurements using homogenates of the other parts of the lungs.

### Measurement of Airway Responsiveness to Histamine

Lung function was assessed by online measurement of pleural pressure (P_pl_) under conscious and unrestrained conditions^53^. In short, a small fluid-filled latex balloon catheter was surgically implanted inside the thoracic cavity and connected to a pressure transducer (TXX-R; Viggo-Spectramed, Bilthoven, The Netherlands) via an external saline-filled cannula. P_pl_ was continuously measured using an online computer system. Changes in P_pl_ are linearly related to changes in airway resistance and serves as a sensitive index for stimulus-induced bronchoconstriction^53^.

Histamine provocations were performed by inhalation of stepwise increasing concentrations of histamine (Sigma, H-7250) in saline (0, 25, 50, 75, 100, 125 and 150 μg/ml). Solution were nebulized for maximally 3 minutes with intervals of 7 minutes, until the P_pl_ was increased by more than 100% above baseline for at least 3 consecutive minutes. The provocation concentration of histamine causing a 100% increase in P_pl_ (PC100) was derived by linear interpolation of the concentration-P_pl_ curve and used as an index for airway responsiveness. Animals were habituated to the experimental conditions as previously described^53^.

### Neutrophil Counting in BAL Fluid and Lung Tissue

BAL was performed as previously described^54^. After anesthesia with pentobarbital (Euthasol 20% i.p.), the trachea was exposed and cannulated, and the lungs were gently lavaged using 5 ml of sterile saline (37 °C), followed by three subsequent aliquots of 8 ml of saline. The recovered BALFs were kept on ice and centrifuged at 200 rcf for 10 min at 4 °C. Pellets were re-suspended into a final volume of 1 ml phosphate-buffered saline (PBS), and a CASY cell counter (Model TT; Innovatis, Reutlingen, Germany) was used to count total cell numbers. For neutrophil determination, cytospin preparations were stained with May-Grünwald and Giemsa stain^54^. Cell differentiation was performed by counting at least 400 cells in duplicate.

Tissue nonspecific alkaline phosphatase (TNAP) staining was used to identify neutrophils on frozen lung sections^26^,^55^. Sections were rinsed in a TRIS-base buffer (pH 7.6) for 2 min, and then incubated for 5 min in a TRIS-base buffer (pH 9.0) containing 1 mg/ml naphthol AS-BI phosphate (Sigma, N2125) and 1 mg/ml Fast Red TR Salt hemi (zinc chloride) salt (Sigma, F8764). Sections were rinsed in a TRIS-base buffer (pH 7.6) for 2 min, counterstained with haematoxylin (Sigma, GHS3) for 1 min, and mounted in Kaiser’s glycerol gelatin. Airway neutrophils were counted in the adventitia and sub-mucosa, and expressed as the number of positively stained cells per mm basement membrane length.

### CBS and Nrf2 Immunohistochemistry

After fixation in acetone, guinea pig lung sections were blocked in 3% hydrogen peroxide (H_2_O_2_) for 30 min, followed by 1 hour blocking in 10% serum (normal rabbit serum (Dako, X0902) for CBS, normal goat serum (Dako, X0907) for Nrf2). Sections were then incubated with primary antibodies (Santa-Cruz, sc-271886, 1:50 for CBS; Abcam, ab31163, 1:100 for Nrf2) for 1 hour at room temperature. Primary antibodies were visualized using horseradish peroxidase-labelled secondary antibodies (1:100) and diaminobenzidine. Sections were counterstained with haematoxylin (Sigma, GHS3) for 1 min and mounted in Kaiser’s glycerol gelatin.

### CBS and Nrf2 Western Blotting

Guinea pig lung homogenates were prepared by pulverizing lung tissue in liquid nitrogen, followed by ultra-sonication in RIPA buffer (composition: 50 mM Tris, 150 mM NaCl, 0.1% sodium dodecyl sulfate, 0.5% sodium deoxycholate, 1% nonyl phenoxypolyethoxylethanol), supplemented with 1 mM Na_3_VO_4_, 1 mM NaF, 1.06 mg/ml β-glycerolphosphate, 10 μg/ml apoprotein, 10 μg/ml leupeptin, and 7 μg/ml pepstatin A. Lysates were centrifuged at 12000 rcf for 20 min at 4 °C, and supernatants were collected.

Protein content was determined using the Pierce BCA protein assay (ThermoFisher, 23225). Equal amounts of protein were separated on a 10% polyacrylamide gel, transferred onto nitrocellulose membranes, blocked with 1x Roti block reagent, and incubated overnight with primary antibodies (Santa-Cruz, sc-271886, CBS: 1:100; Abcam, ab31163, Nrf2: 1:1000; Santa-Cruz, sc-47724, GAPDH: 1:400) at 4 ^o^C. After washing, membranes were incubated with horseradish peroxidase-labelled secondary antibodies. Protein bands were visualized using Western lightning plus ECL (PerkinElmer, NEL105001EA) and quantified using Image J 1.48v. CBS and Nrf2 were normalized to GAPDH.

### MDA Measurement

MDA concentrations in guinea pig lung homogenates was measured by the thiobarbituric acid reactive substances assay^56^. Lung lysates were mixed with 10% trichloroacetic acid (Sigma, T9159; 1:1, v/v) and centrifuged at 2,200 x g for 15 min for protein removal. Samples were mixed with 6.7 g/l thiobarbituric acid (Sigma, T5500; 1:1, v/v) and heated at 95 °C. MDA levels were determined by measuring absorption at 550 nm. The standard curve was made by measuring a series of gradually diluted 1,1,3,3,-tetramethoxypropane (Sigma, 108383) solutions. MDA levels in lung homogenates were expressed as μmol MDA per g protein.

### H_2_S Measurement

H_2_S levels were determined by measuring sulfide levels in alkaline liquid solutions. Equal amounts of serum or cell culture supernatant and SOAB buffer (390 mM sodium salicylate, 9.2 mM ascorbic acid, 531 mM NaOH in H_2_O) were mixed. The resulting sulfide levels were measured using an ISM-146S electrode (Lazar, Los Angeles, USA) in combination with a 6230N mV meter (Jenco, San Diego, USA).

### ASM Cell Culture

Three human airway smooth cell lines, immortalized by human telomerase reverse transcriptase (hTERT)^57^ were used for all the experiments. The primary cultured human airway smooth cells used to generate each hTERT immortalized cell line were prepared as described previously^57^. Informed consent was obtained from all subjects. All procedures were in accordance with the relevant guidelines and approved by the Human Research Ethics Board of the University of Manitoba. The cells were maintained in Dulbecco’s modified Eagle’s medium (DMEM, Life technologies, 11965-092) containing heat-inactivated fetal bovine serum (10% vol/vol), streptomycin (50 U/ml), penicillin (50 mg/ml) in a humidified atmosphere at 37 °C in air/CO_2_ (95%:5% vol/vol).

### Cigarette Smoke Extract Preparation

Cigarette smoke extract (CSE) was freshly prepared by pumping the smoke from two combusted 3R4F research cigarettes (Reference Cigarette Program, University of Kentucky) through 25 ml of serum-free DMEM^10^,^28^. This was designated as 100% CSE.

### IL-8 Measurement

hTERT-ASM cells were plated on 24-well plates. After grown to confluence, cells were treated with the indicated concentrations of the Sul-121 in the absence and presence of 15% CSE for 24 hours in serum-free DMEM. Culture medium was collected to measure IL-8 concentrations using an IL-8 enzyme-linked immunosorbent assay (ELISA) kit (Pelikine, M1918) according to the manufacturer’s instructions. Fenoterol (Boehringer Ingelheim, 217-742-8, 1 μM) treatment was used as a positive control. Cells were trypsinized for trypan blue cell counting to determine cell viability^10^. Data represent from 7–24 experiments.

### Nrf2 and p65 Immunofluorescence

ASM cells were plated on cover slips in 12-well plate. After grown to confluence, cells were treated with 300 μM Sul-121, with or without 15% CSE or 10 μg/ml LPS (Sigma, L-2880), for 2 hours. Cells were then fixed with a solution containing 4% paraformaldehyde and 4% sucrose for 15 min at room temperature, followed by treatment with 0.3% Triton X-100 for 5 min at room temperature. Cells were blocked for 1 hour at room temperature with PBS containing 5% bovine serum albumin and 2% donkey serum. Cells were then incubated overnight at 4 °C with primary antibodies against Nrf2 (Abcam, ab31163, 1:100) and p65 (Cell Signaling, #3033S, 1:20). The next day, cells were washed with PBS and incubated with secondary antibodies (1:500) for 1 hour at room temperature. After wash with PBS, nuclei were stained with Hoechst (Invitrogen, H3570, 1:10000) for 5–10 sec, immediately followed by two quick and four 10 min washing steps with ultra-pure water. After staining, coverslips were mounted using ProLong® Gold Antifade Mountant reagent (Life Technologies, P36930) and imaged using an Olympus AX70 microscope equipped with digital image capture system (ColorView Soft System with Olympus U CMAD2 lens, Olympus Corporation, Tokyo, Japan). The background corrected fluorescence measurements were performed with Image J 1.48v^58^. Data represent from 4–5 experiments.

### ROS Measurement

The 7′-dichlorofluorescein-diacetate (DCF-DA) fluorescence method was used for the determination of ROS level. 6-carboxy-2′,7′-dichloro-dihydrofluorescein diacetate, di(acetoxymethyl ester) (Carboxy-H2DCF-DA, Life technologies, C-2938, 0.1 μM) was incubated for 40 min with 300 μM Sul-121 in the absence and presence of H_2_O_2_ (Merck KGaA, 1.07209.0250, 2 mM) or CSE (15%). The ROS level was measured by the intensity of DCF emission at 525 nm (excitation 503 nm). Alternatively, dihydroethidium (DHE, Life technologies #D11347, Carlsbad, CA, 5 μM) was dissolved in DMEM and supplemented with SUL-121 (1–100 μM). Hydrogen peroxide (H_2_O_2_, MerckMillipore #107209, Darmstadt, Germany, 5 μM) was added as reactive oxygen donor and samples were incubated at room temperature for 1 hour. Fluorescence was recorded on a Varioskan spectrofluorometer (ThermoScientific, Waltham, MA) at Ex/Em 488/525 nm and 518/605 nm for DCF and DHE, respectively. Data represent 3–12 experiments.

To examine the production of ROS, ASM cells were plated on 96-well plates. After grown to confluence, cells were treated with 300 μM Sul-121 in the absence and presence of 0.1 μM PMA (Sigma, P-8139) as control^59^ for 2 hours. 10 mM NAC (Sigma, A9165) served as positive control. After removal of the supernatant, cells were incubated with 0.1 μM carboxy-H2DCFDA for 1 hour and ROS production was measured as described above. Data represent 4 experiments.

### Statistics

Data represent means ± SEM, from *n* experiments. Statistical significance of differences was evaluated by one-way or two way ANOVA with Bonferroni post-hoc tests, or by two tailed Student’s t-test using Prism 5 software. Pearson’s correlation tests were also performed by using Prism5. Differences were considered to be statistically significant when p < 0.05.

All the figures and pictures were created by authors of this paper.

All *in vivo* protocols described in this study were approved by the University of Groningen (Groningen, The Netherlands) Committee for Animal Experimentation. All the methods were carried out in accordance with the approved guidelines.

For experiments involving human samples, informed consent was obtained from all subjects. All procedures were in accordance with the relevant guidelines and approved by the Human Research Ethics Board of the University of Manitoba.

## Additional Information

**How to cite this article**: Han, B. *et al.* The novel compound Sul-121 inhibits airway inflammation and hyperresponsiveness in experimental models of chronic obstructive pulmonary disease. *Sci. Rep.* **6**, 26928; doi: 10.1038/srep26928 (2016).

## Supplementary Material

Supplementary Information
