# Supplementary material for: The novel compound Sul-121 inhibits airway inflammation and hyperresponsiveness in experimental models of chronic obstructive pulmonary disease
Source: Sci Rep. 2016 May 27;6:26928. doi: 10.1038/srep26928 (PMC4882609; doi:10.1038/srep26928)

The novel compound Sul-121 inhibits airway inflammation and hyperresponsiveness in experimental models of chronic obstructive pulmonary disease

Bing Han[1,2], Wilfred J. Poppinga[1,2], Haoxiao Zuo[1,2], Annet B. Zuidhof[1], I. Sophie T. Bos[1], Marieke Smit[1], Pieter Vogelaar[3], Guido Krenning[4], Robert H. Henning[5], Harm Maarsingh[6], Andrew J. Halayko[7], Bernard van Vliet[3], Stef Stienstra[3], Adrianus Cornelis van der Graaf[3], Herman Meurs[1,2], Martina Schmidt[1,2]

[1]University of Groningen, Department of Molecular Pharmacology, Groningen, the Netherlands
[2]GRIAC research institute, University of Groningen, University Medical Center Groningen, the Netherlands
[3]Sulfateq B.V. Groningen, the Netherlands
[4]University of Groningen, University Medical Center Groningen, Dept. Pathology and Medical Biology, Laboratory for Cardiovascular Regenerative Medicine, Groningen, the Netherlands
[5]University of Groningen, Department of Clinical Pharmacy and Pharmacology, Groningen, the Netherlands
[6]Palm Beach Atlantic University, Lloyd L. Gregory School of Pharmacy, Department of Pharmaceutical Sciences, West Palm Beach, FL, USA
[7]Department of Physiology and Pathophysiology, University of Manitoba, Winnipeg, Manitoba, Canada

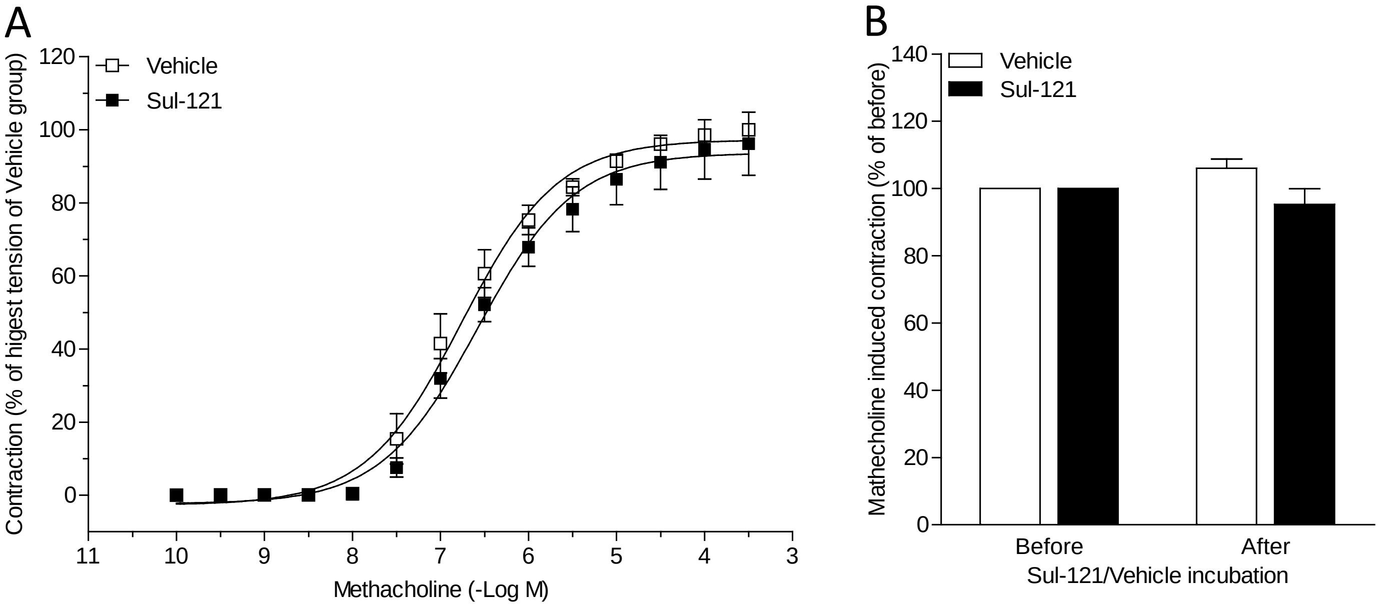

Bovine tracheal smooth muscle (BTSM) strips were prepared and mounted for isometric recording as previously described[57,60]. To study effects of Sul-121 on muscarinic receptor agonist-induced contraction, BTSM strips were incubated with 30 µM Sul-121 or vehicle for 30 min, followed by cumulative dosing of the indicated methacholine concentrations (A). To analyze Sul-121 effects on contractile muscle function tone, BTSM strips were first contracted by 0.3 µM methacholine, followed by washout, incubation of 600 µM Sul-121 for 2 hours, and a subsequent washout. BTSM strips were contracted again with 0.3 µM methacholine. Methacholine-induced contractions before/after Sul-121 incubation were compared (B). N=3-4.

57. Dekkers, B. G., Schaafsma, D., Nelemans, S. A., Zaagsma, J. & Meurs, H. Extracellular matrix proteins differentially regulate airway smooth muscle phenotype and function. Am. J. Physiol. Cell. Mol. Physiol. 292, L1405–13 (2007).
60. Roscioni, S. S. et al. cAMP inhibits modulation of airway smooth muscle phenotype via the exchange protein activated by cAMP (Epac) and protein kinase A. Br. J. Pharmacol. 162, 193–209 (2011).

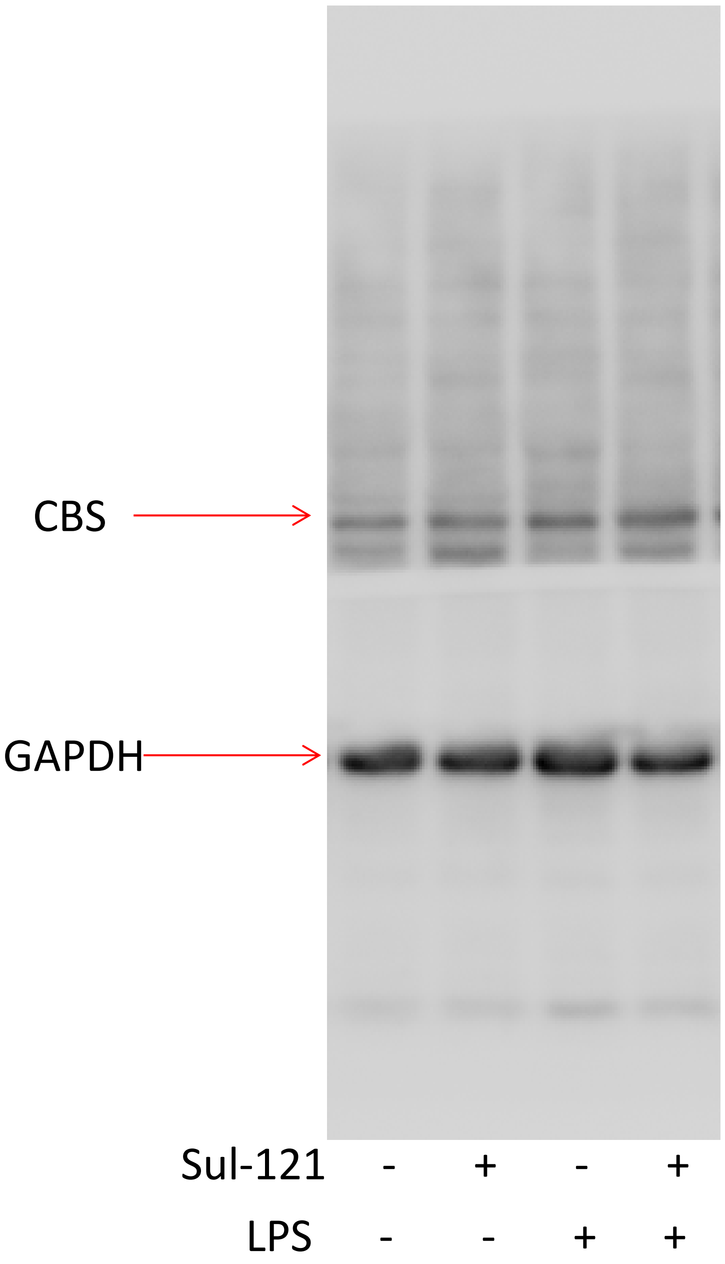

Western blotting has been performed under the same experimental conditions. The blot was cut to incubate with anti-CBS and anti-GAPDH antibodies separately. Subsequently, the blots were developed at the same exposure time.

Western blotting has been performed under the same experimental conditions. The blot was cut to incubate with anti-Nrf2 and anti-GAPDH antibodies separately. Subsequently, the blots were developed using different exposure times for Nrf2 and GAPDH.

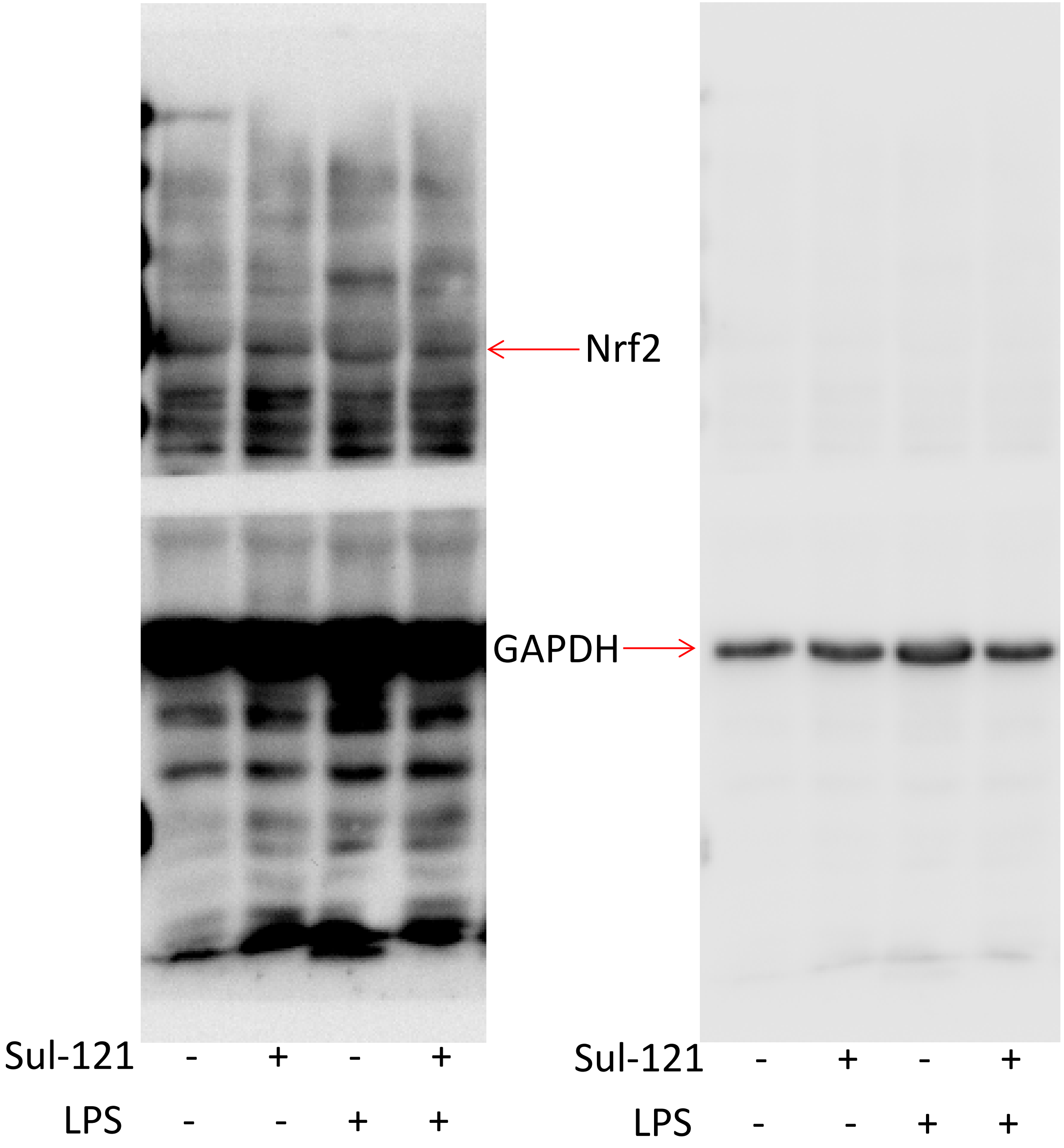

Supplement: Supplementary Information [file srep26928-s1.pdf]
